# Supplementary material for: Complexome profiling of the Chlamydomonas psb28 mutant reveals TEF5 as an early PSII assembly factor
Source: Plant Cell. 2025 Mar 17;37(6):koaf055. doi: 10.1093/plcell/koaf055 (PMC12164590; doi:10.1093/plcell/koaf055)
Supplement: koaf055_Supplementary_Data [file koaf055_supplementary_data.zip › psb28_interactive.zip!/plots/intensity/qualityPlots/peptides/_Cre17.g725400.t1.2.html]

### Cre17.g725400.t1.2

Trivial name:   
SeqCoverage [median]: 12.10  
Mass: 13.83 kDa  
  
 

| Sequence | MissedCleavage | Mass [Da] | wtIntensity | muIntensity | Score |
| --- | --- | --- | --- | --- | --- |
| DGTLPEGESSGHGHH | 0 | 1515.63 | 12752.28 | 12529.02 | 132.50 |
|  |  |  | 12752.3 | 12529.0 |  |
